# The Accuracy and Precision of Position and Orientation Tracking in the HTC Vive Virtual Reality System for Scientific Research

**DOI:** 10.1177/2041669517708205

**Published:** 2017-05-18

**Authors:** Diederick C. Niehorster, Li Li, Markus Lappe

**Affiliations:** Institute for Psychology, University of Muenster, Germany; Neural Science Program, New York University Shanghai, China; Department of Psychology, The University of Hong Kong, Hong Kong; Institute for Psychology, University of Muenster, Germany; Otto-Creutzfeldt Center for Cognitive and Behavioural Neuroscience, University of Muenster, Germany

**Keywords:** virtual reality, head mounted display, position tracking, data quality, natural vision

## Abstract

The advent of inexpensive consumer virtual reality equipment enables many more researchers to study perception with naturally moving observers. One such system, the HTC Vive, offers a large field-of-view, high-resolution head mounted display together with a room-scale tracking system for less than a thousand U.S. dollars. If the position and orientation tracking of this system is of sufficient accuracy and precision, it could be suitable for much research that is currently done with far more expensive systems. Here we present a quantitative test of the HTC Vive’s position and orientation tracking as well as its end-to-end system latency. We report that while the precision of the Vive’s tracking measurements is high and its system latency (22 ms) is low, its position and orientation measurements are provided in a coordinate system that is tilted with respect to the physical ground plane. Because large changes in offset were found whenever tracking was briefly lost, it cannot be corrected for with a one-time calibration procedure. We conclude that the varying offset between the virtual and the physical tracking space makes the HTC Vive at present unsuitable for scientific experiments that require accurate visual stimulation of self-motion through a virtual world. It may however be suited for other experiments that do not have this requirement.

## Introduction

For the past 20 years, researchers using virtual reality (VR) techniques have made great strides in examining human vision of the actively moving and exploring observer. VR setups, for instance, afford participants to walk through computer-controlled environments that can be manipulated to test hypotheses that are hard to examine systematically in the real world (e.g., Fink, Foo, & Warren, 2009; Warren, Kay, Duchon, Zosh, & Sahuc, 2001). Furthermore, the perception of the freely moving observer may be different in important ways from the perception of the seated observer as studied in the lab. Experiments have revealed differences in depth perception between static and moving observers, for example, in the perception of object stationarity and structure from motion (Tcheang, Gilson, & Glennerster, 2005; Wexler, Panerai, Lamouret, & Droulez, 2001), and size constancy (Combe & Wexler, 2010). As such, calls have long been made to take the study of vision and other behavior out of the lab and study the perception of the moving observer of everyday life (e.g., recently Scarfe & Glennerster, 2015; Wexler & Van Boxtel, 2005).

The VR equipment required for these experiments typically consists of a head mounted display (HMD) and a position and orientation tracking system. This hardware has long been very expensive with costs ranging from multiple tens of thousands to close to a hundred thousand U.S. dollars, placing it out of reach for many researchers whose work could benefit from using VR technology. Recently, VR technology is starting to become available in the consumer gaming market, leading to products such as the Oculus Rift and the HTC Vive that sell for less than 1,000 U.S. dollars. While the Oculus Rift available at the time of this article is designed for movement in only a small area, the HTC Vive is designed to track an observer who freely moves through a space of up to 4 × 4 m. As a complete HMD and position and orientation tracking system at this price point, the Vive could enable a much larger number of researchers to access VR technology and employ it to study human vision and behavior in naturalistic unconstrained environments.

Our subjective experience when playing a series of computer games developed for the Vive indicates that the tracking of the system appears stable and fast, making the Vive an interesting candidate VR system for scientific research. However, for the HTC Vive to be able to fulfil the promise of an accessible VR system for this purpose, it is important that its position and orientation tracking is of sufficient accuracy (low offsets between reported and actual position and orientation in the tracking space) and precision (jitter in the reported position and orientation measures) to enable the presentation of naturalistic stimuli that accurately simulate the motion of the observer through a virtual space. Furthermore, the latency between physical movement of the headset and the corresponding update of the Vive’s display needs to be sufficiently low to prevent the virtual environment from not appearing stable in three-dimensional (3D) space (*swimming*, e.g., Allison, Harris, Jenkins, Jasiobedzka, & Zacher, 2001; Jerald & Whitton, 2009) and to prevent motion sickness (e.g., Kinsella, Mattfeld, Muth, & Hoover, 2016). The only existing assessments of consumer-level VR systems (Chessa, Maiello, Borsari, & Bex, 2016; Kim, Chung, Nakamura, Palmisano, & Khuu, 2015) however only investigated their suitability for research with seated observers, and only dealt with issues of vection and presence. In this study, we quantitatively examined the performance of the Vive’s position and orientation tracking output, as well as its end-to-end system latency from physical movement to display update. To ensure that our results accurately represent the Vive’s abilities, we have run our tests using two identical Vive systems, and, for comparison, on a research-grade WorldViz Precision Position Tracking (PPT) system. We ran tests in two differently sized tracking areas, one matching our experimental setup of 8 by 4 m and the other matching the Vive manufacturer’s suggested maximum tracking space of 4 by 4 m. Our main findings were consistently observed in all these tests. Our work for the first time tested the suitability of consumer-level VR systems for scientific research with walking observers.

## HTC Vive Overview

The Vive consists of a headset, two controllers, and two infrared laser emitter units. The headset covers a nominal field of view of about 110° (approximately 90° per eye) through two 1080 × 1200 pixel displays that are updated at 90 Hz. As such, the Vive’s pixel density is about 12 pixels/°, which means individual pixels can easily be seen when looking for them. This pixel density is similar to that of other current headsets and may limit the use of the Vive in psychophysical experiments. The Vive’s tracker operates on a so-called *inside-out principle*, where no external cameras are needed. Instead, the Vive’s tracking technology uses two laser emitters, called Lighthouses. These two boxes alternatingly send out horizontal and vertical infrared laser sweeps spanning 120° in each direction. On the surface of the Vive’s headset and the controllers are photodiodes that indicate when the laser hits them. The difference in time at which the various photodiodes are hit by the laser allows recovery of the position and orientation of the headset. The Vive headset’s current tracked position and orientation are however updated primarily by inertial measurement units in the headset through dead reckoning (path integration) because that allows for much higher update rates. The lighthouse units serve to limit and correct for the build-up in error that is inherent in dead reckoning based on inertial measurements, and it does so at a rate of 120 Hz (Kreylos, 2016). Nonetheless, when the view of both lighthouses is lost, the Vive’s screen turns gray and the Vive reports unchanging position and orientation values instead of continuing to update based on the measurements from the inertial measurement units. The Vive furthermore has a obliquely downward-facing camera on its front that delivers a 1280 × 720 pixel image at 60 Hz.

While games played with the Vive allow physical movement within a play area that is limited to 4 × 4 m, there are no set limits to the range over which the Vive delivers position and orientation data. We used several setups in the tests reported here and found that subjectively, tracking quality of all our setups, including the largest where the lighthouse units were placed 7.45 m apart, seemed equally good. The largest setup delivered tracking data over a range of about 8 m along the long axis of our room. After placement of the two lighthouse units, setup involves running through a calibration routine provided by the Vive’s software package. During this procedure, the two Vive controllers are placed close together on the floor in the center of the track space so that the system can calibrate where the floor is and how it is oriented. Furthermore, at this stage the “play area” is set up, defining the origin and orientation of the Vive’s tracking coordinate system in the *X-Z* plane.

## General Methods

### Apparatus

**Figure 1.**
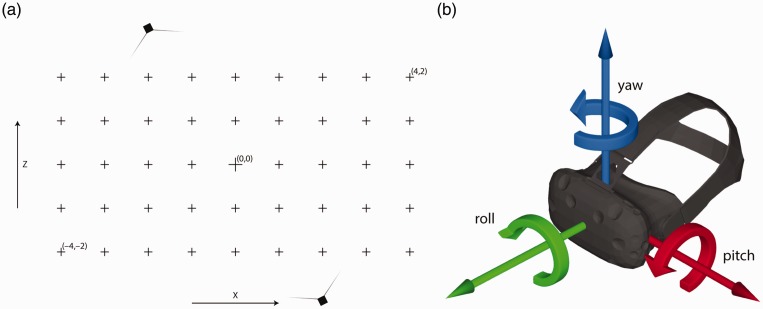
(a) Top view of the measurement setup. The crosses indicate the grid points that were drawn on the floor, and the black boxes indicate the lighthouses units. (b) Schematic representation of the axis conventions used in this study.

All measurements were taken in an 11 × 7 m room with a 3.2 m high ceiling, lighted by fluorescent lighting, with no reflective surfaces and no exposure to natural lighting. The floor was level to gravity as verified with a spirit level. In the center of this room, an 8 × 4 m Cartesian grid was drawn on the floor using string and chalk, with grid lines spaced by 1 m (see Figure 1(a)). Average positioning error of grid points was 1.7 cm (*SD*: 0.9 cm), with 90% below 3 cm and 80% below 2 cm. The origin of this grid is defined during the setup of the Vive’s tracking system when the play area is defined by placing a Vive controller at the limits of safe walking space. For the purpose of this test, we placed the Vive controller at grid locations (−3, −2), (3, −2), (3, 2), and (−3, 2), thereby placing the origin at the center of the grid shown in Figure 1(a).

The lighthouses were mounted on stable tripods at a height of 2.45 m from the ground and were connected to each other with the provided sync cable. For the five tests reported in this article, we have placed the lighthouses at multiple distances. In Tests 1 to 3, the two lighthouse units were placed 7.45 m apart to test the suitability of the Vive for VR experiments that require a relatively large walking area. Specifically, the lighthouse units were placed at approximately (−2, 3.15) and (2, −3.15), at either side of the grid (indicated by the boxes in Figure 1(a)). The Vive software detected the distance between the two lighthouses with an error of only 3 cm. Given their large distance from each other and to ensure that they were pointed reasonably far into the tracking area, the lighthouse units were rotated 7.2° outward away from the center of the room and pointed downward at an angle of 25°, which is slightly less than the angle recommended by the manufacturer (30°–45°). In Test 4, we used a setup within the maximum operating range of 5.5 m suggested by the manufacturer by placing the lighthouse units 5 m apart, at (−2, 2) and (1, −2). For this test, the lighthouse units faced directly toward each other and pointed downward at an angle of 37.5°, which is in the middle of the range recommended by the manufacturer. In Test 5, to ensure that our results are not due to a fault in the specific Vive system that we had acquired, we replicated Test 4 with a second Vive system.

In Tests 1 to 4, the Vive headset was mounted rigidly and level to the floor on a stable tripod with a heavy cast-iron foot. Given the dimensions and shape of the foot of this tripod, error in manual placement on the grid points was smaller than 2 cm. On this mount, the center of the Vive’s front was 1.65 m from the floor, and the field of view of none of its sensors facing parallel to the floor or upward was occluded by the mount. In Test 5, measurement was done with a different stand that allowed for an error in manual placement on the grid points of smaller than 1 cm. On this stand, the center of the Vive’s front was 1.55 m above the ground. To this stand, we also rigidly mounted two infrared-emitting markers at 19.5 cm distance from each other to enable position measurement with a research-grade Worldviz PPT-X optical tracking system. Last, we mounted an Intersense InertiaCube4 for orientation measurement. This setup allowed us to make simultaneous measurements with the Vive and this research-grade motion tracking system, to allow for a direct comparison of the performance of these systems. The data from the research-grade system indicated that the stand was placed with a 3° standard deviation in yaw orientation (including noise in the Intersense’s measurements).

The Vive was connected to the measurement computer through a custom-built 20 m cable providing an HMDI connection, a USB 2 connection, and power. Subjectively, tracking appeared stable in this configuration, and VR games were playable with no visible tracker artifacts. Presence during gameplay was good due to solid nonjittery tracking.

The position and orientation data provided by the Vive were acquired through the SteamVR plugin of Vizard 5.5 (Worldviz) at the Vive’s 90 Hz refresh rate. The video feeds of the PPT system’s cameras were processed on a separate computer in PPT Studio 2008 and made available over a private wireless Ethernet network via the virtual reality peripheral network (VRPN) protocol. The InertiaCube was directly connected to the measurement computer by USB cable. The data from the PPT and InertiaCube were acquired through the VRPN 7 and the Intersense plugins, respectively, of Vizard 5.5 (Worldviz) at the Vive’s 90 Hz refresh rate. To recover *X*, *Y*, and *Z* positions as well as yaw and roll angles, the OpticalHeading plugin of Vizard 5.5 was used. This plugin takes the data of the two PPT markers to create an optical compass whose orientation is merged with that indicated by the InertiaCube to counteract possible drift in the output of the InertiaCube. Pitch was provided directly by the InertiaCube. After the stand with the Vive mounted on it was placed at a location to measure, it was left for 5 s to make sure it was fully static. The trigger of the Vive controller was then pulled, starting the capturing of 2 to 3 s of position and orientation data. The axis conventions used for the orientation measures in this study are shown in Figure 1(b).

### Data Analysis

We assessed the accuracy and precision of the position and orientation measures reported by the Vive at each location in our grid. Specifically, for accuracy we looked at whether the *X-Z* positions reported by the Vive were regularly spaced along the grid and did not exhibit signs of warping, whether reported height above the ground was stable across space and whether reported pitch and roll orientation of the Vive were stable across space. For precision, at each point on the grid we analyzed both the size of frame-to-frame jumps in the position and orientation data reported by the Vive, and the spatial extent of the *X-Z* position noise. All analyses were performed in Matlab (the Mathworks). The data acquisition scripts, data analysis scripts, and the data files are freely available from http://dx.doi.org/10.5281/zenodo.569884.

#### Accuracy of reported spatial position

To assess accuracy of the spatial position data reported by the Vive, we plot the mean measured *X-Z* position for each grid point. The mean of the height reported by the Vive at each grid point is further visualized in a heat map format.

#### Accuracy of reported pitch and roll orientation

The height from the ground reported by the Vive at each of the grid points indicated that the ground plane reference it used was tilted away from the true ground plane. Due to this tilt, we expected that the pitch and roll values reported by the Vive would be systematically offset from their true value. We tested whether the reported orientation values at each grid point were internally consistent with the Vive’s tilted reference plane and the yaw orientation of the Vive with respect to this plane. If this is the case, a calibration procedure could determine the orientation of the Vive’s reference plane and correct for it.

The orientation of the Vive’s reference plane was assessed by finding the 3 × 3 rotation matrix R describing the rotation from the physical ground to the Vive’s reference plane. Given a set of *n* physical measurement positions $P_{w}^{i}=[X_{w}^{i},Y_{w}^{i},Z_{w}^{i}{]}^{T}$, where $(X_{w}^{i},Z_{w}^{i})$ are physical grid locations in the room and *Y_w_* is the Vive’s mounting height of 1.65 m, and the corresponding set of *n* 3D position measurements reported by the Vive $P_{v}^{i}=[X_{v}^{i},Y_{v}^{i},Z_{v}^{i}{]}^{T}$, this rotation matrix is given as follows (Arun, Huang, & Blostein, 1987). We first calculated the 3 × 3 matrix $H_{\mathrm{wv}}$ of covariances between the data along each of the components in *P_w_* and *P_v_* as

$$H_{\mathrm{wv}}=\sum_{i=1}^{n}(P_{w}^{i}-\mathrm{centroid}(P_{w}))(P_{v}^{i}-\mathrm{centroid}(P_{v}){)}^{T},$$

where $\mathrm{centroid}(P_{x})$ denotes the arithmetic mean of a set of positions $P_{x}^{i}$. After performing singular value decomposition of the matrix

$$H_{\mathrm{wv}}=U\Sigma V^{T}$$

which yields the left- and right-singular unitary matrices U and $V^{T}$ and the matrix Σ containing the singular values of $H_{\mathrm{wv}}$ along its diagonals, R is then given by

$$R={\mathrm{VU}}^{T}.$$

The inverse of this rotation matrix, $R^{-1}$, gives the rotation required to convert the Vive’s reported orientation measures to the true orientation in physical space. A complete calibration that corrects for both offsets in orientation and position in the values reported by the Vive is then given by

$$P_{w}=R^{-1}P_{v}+t$$

where *t* is a translation vector given by

$$t=-R^{-1}\mathrm{centroid}(P_{v})+\mathrm{centroid}(P_{w}).$$

The resulting rotation matrix $R^{-1}$ and translation vector *t* form the best possible calibration between the Vive tracker’s space and the true physical space, in the least squares sense, minimizing the quantity:

$$S=\sum_{i=1}^{n}\|R^{-1}P_{v}^{i}+t-P_{w}^{i}{\|}^{2}$$

While this calibration method does not correct for any scaling or shearing transformations, this did not appear necessary.

We then assessed whether the yaw, pitch, and roll orientations reported at each grid location by the Vive were internally consistent with its tilted and rotated reference plane as determined with the above method. Given a yaw orientation reported by the Vive, we calculated the predicted pitch and roll orientations at each measurement position as follows. For each measurement at each grid location, we first rotated the Vive’s reference frame by the yaw angle θ reported by the Vive, ${\mathrm{RR}}_{\underline{\theta }}$, where $R_{\underline{\theta }}$ is a rotation matrix describing a yaw rotation by angle θ. We then decomposed the resulting rotation matrix into Euler angles to recover the expected pitch and roll values and compared these to the pitch and roll values reported by the Vive.

#### Sample-to-sample jitter

Tracker jitter was assessed by means of the root mean square (RMS) of the change in the position and orientation values reported by the Vive. RMS indicates the size of jumps that are made from frame-to-frame in the tracker output, and in that sense indicates the velocity of the noise in the position and orientation measures. The larger the RMS, the more visible the jitter artifacts become. RMS for a data segment of *n* samples is given by:

$${\text{RMS}}_{m}=\sqrt{\frac{1}{n}\sum_{i=1}^{n-1}\Delta m_{i}^{2}}$$

where $\Delta m_{i}$ is the difference between samples *i* and i+1 of the measure *m* under consideration.

#### Spatial extent of noise in reported position in the *X-Z* plane

To assess spatial spread of the reported position, the bivariate contour ellipse area (BCEA) method common in the eye-tracking literature (see e.g., Blignaut & Beelders, 2012; Crossland & Rubin, 2002) was used. The BCEA is a measure of the area covered by a collection of samples. It is different from the RMS measure in that, for instance, a slow drift would yield a low RMS value but could yield a large BCEA value if it covers a large spatial area over time. BCEA is calculated as follows:

$${\text{BCEA}}_{x}=2k\pi \sigma_{X}\sigma_{Z}\sqrt{1-\rho^{2}}$$

where $\sigma_{X}$ and $\sigma_{Z}$ are the standard deviations of measured positions along the *X* and *Z* axes, respectively, and ρ is the product-moment correlation of the measured positions along these axes. *k* depends on the probability area (P) indicating that the BCEA ellipse is sized such that the measured positions are within its contours for *P*% of the time if they are bivariate-normally distributed. *k* is given by k=-log(1-P). *P* was set to 0.68 (1 *SD*) for this study, consistent with previous work (e.g., Crossland & Rubin, 2002).

To further examine whether the noise in the *X-Z* plane is isotropic, we calculated the aspect ratio and orientation of the BCEA ellipse. Factorization of the 2 × 2 covariance matrix of the measured positions along the *X* and *Z* axis

$$V_{\mathrm{XZ}}=[\sigma_{X}^{2}\rho \sigma_{X}\sigma_{Z}\rho \sigma_{X}\sigma_{Z}\sigma_{Z}^{2}]$$

through eigenvalue decomposition

$$V_{\mathrm{XZ}}=Q\Lambda Q^{-1}$$

yields the 2 × 2 matrix Q whose columns are the eigenvectors of $V_{\mathrm{XZ}}$ and diagonal matrix $\Lambda =\text{diag}(\lambda_{1},\lambda_{2})$, where $\lambda_{1}$ and $\lambda_{2}$ are the eigenvalues of $V_{\mathrm{XZ}}$. The eigenvalues correspond to the squared relative lengths of the principle axes of the BCEA ellipse when k=1. As such, the aspect ratio (AR) of the BCEA ellipse is given by:

$$\mathrm{AR}=\sqrt{\frac{\lambda_{1}}{\lambda_{2}}}$$

The orientation θ of the major axis of the BCEA ellipse is given by:

$$\theta =\tan^{-1}\frac{q_{21}}{q_{11}}$$

Because only the orientation, aspect ratio, and relative size of the BCEA ellipses were of interest in the visualizations we present below, the ellipses were scaled by a common factor to provide a clear visualization.

## Results

### 1. Accuracy Along the Grid

**Figure 2.**
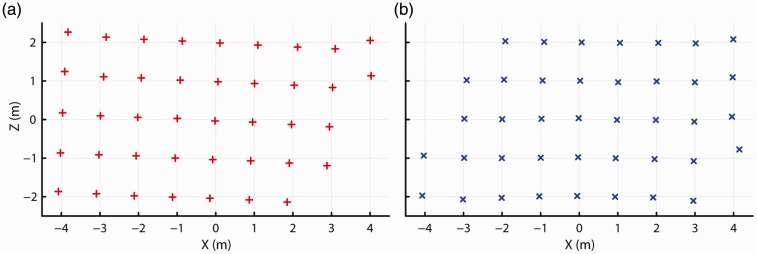
Recorded *X-Z* position (a) for when the headset faced along the positive *X* axis and (b) for when the headset faced along the negative *X* axis.

To assess data quality in the tracked area, we placed the Vive at each of the grid points (see Figure 1(a)) while the headset was facing either along the positive *X* axis, or along the negative *X* axis of the room. At each point, 1 s of data was acquired. Figure 2 shows the mean recorded *X-Z* positions at each grid point. Most of the recorded *X-Z* positions seem to fall along a regular grid, both when the headset faced along the negative and the positive *X* axis of the room. Measures for some of the grid points were not available as tracking was lost there. The grid of measured locations appeared slightly rotated from its physical orientation, perhaps due to small but consistent errors in recorded positions of the Vive controllers during the set up procedure of the tracking space. More importantly, it can be seen that some of the recorded positions at the extremes along the *X* axis appeared to be systematically offset from the rest of the grid. This offset occurred after the Vive headset temporarily lost tracking, possibly due to occlusion as we moved it from one grid point to another.

**Figure 3.**
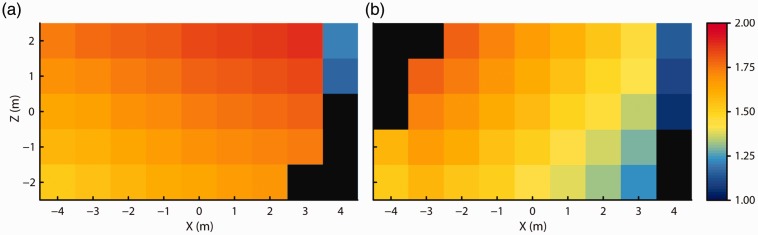
Recorded height at each of the grid locations for (a) when the headset faced along the positive *X* axis and for (b) when it faced along the negative *X* axis. No data could be recorded at the black locations due to loss of tracking.

Figure 3 shows the height above the ground of the headset reported by the Vive at each of the grid locations. It appears that the recorded height systematically varied across space, despite that the headset was physically at the same height above the floor of the room for each measurement. Furthermore, the recorded height was systematically offset at some of the extreme measurement locations along the *X* axis of the room. The locations at which these offsets occurred are the same locations for which the sudden offsets in *X-Z* position are observed in Figure 2. Because significant changes were observed in the reported position measures after tracking was lost, it was important to first collect a dataset in which no intermittent loss of tracking occurred while the Vive was moved along the grid to examine the baseline data quality in the tracked space.

### 2. Data Quality Without Intervening Loss of Tracking

To avoid the problems with sudden offsets in recorded positions, we collected a second dataset in which no intervening loss of tracking occurred while the Vive was moved along the grid. To achieve this, we avoided recording at a few locations at the edge of the grid where data loss was likely to occur. Three seconds of data (270 samples) was acquired at each grid location in one measurement session in which the headset faced along the *X* axis of the room in both directions. This allowed for a test of whether data quality depended on the facing direction of the headset.

**Figure 4.**
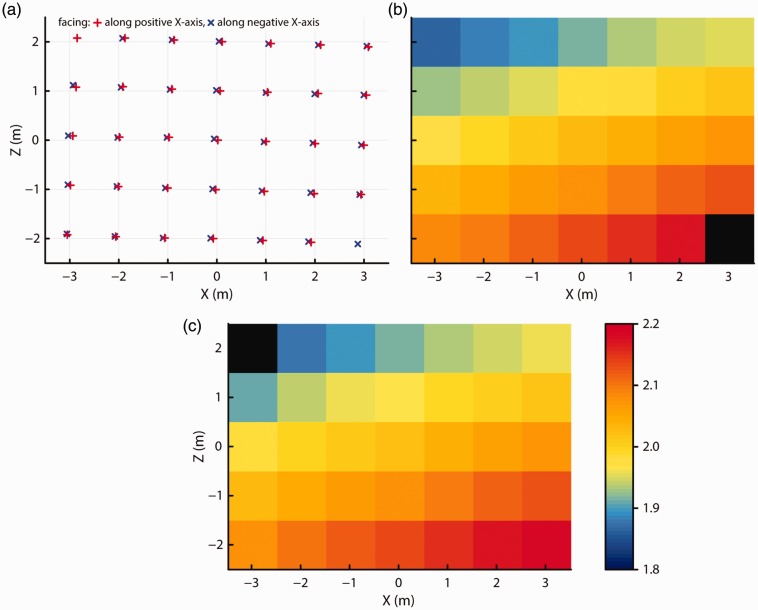
Recorded (a) *X-Z* position for both headset facing directions. (b) and (c) Recorded height at each of the grid locations for (b) when the headset faced along the positive *X* axis, and for (c) when it faced along the negative *X* axis. No data could be recorded at the black locations due to the loss of tracking.

Figure 4 shows recorded *X-Z* position and height at each of the grid locations for both of the facing directions of the headset. As before, the reported locations formed a slightly rotated but regular grid. The position measurements closely coincided at each grid location for both facing directions. Furthermore, the measurements again showed a systematic change across space in reported height above the floor. Height measurements were also consistent for the two headset facing directions.

**Figure 5.**
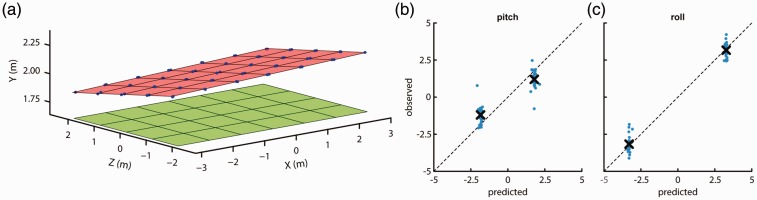
(a) The Vive’s reference plane (red) fit to the 3D measurement locations (blue dots) reported by the Vive. The green plane indicates the plane formed by the physical measurement locations. Observed (b) pitch and (c) roll orientations against the predicted values for a physically level headset based on the Vive’s tilted reference plane. Blue dots indicate individual measurements, and the black cross the mean of these measurements for each headset facing direction.

The systematic change of measured height across the grid suggests that the Vive uses a reference plane for its measurements that is titled with respect to the physical ground plane. If this is the case, the measured roll and pitch angles should also be systematically offset. We investigated this by determining the optimal 3D rotation that transforms the Vive’s measured 3D grid locations into to physical location of the headset by means of a calibration procedure (see Methods section). The plane (Figure 5(a)) was fit to the mean measured 3D positions for all grid locations and both headset facing directions. Next, we predicted what pitch and roll orientations the Vive should report given the tilted reference plane and the Vive’s yaw orientation at each measurement location. The observed pitch and roll orientations are plotted in Figure 5(b) and (c) against their predicted values. Indeed, the observed pitch and roll orientations are both close to the predicted orientations, indicating that the measured height, yaw, pitch, and roll at each grid location are internally consistent with each other. This suggests that although the Vive appears to base its measurements on a reference plane that is tilted away from the physical ground plane, this offset is consistent and could thus be calibrated and corrected for with the same plane fit procedure we used here. However, the systematic offsets in recorded 3D position observed after the loss of tracking in the first recording indicate that the Vive’s reference plane may change after loss of tracking, which would make this calibration approach difficult to implement in practice. We further investigated whether the Vive’s reference plane changes after loss of tracking with separate data recordings reported in Test 3 below.

**Figure 6.**
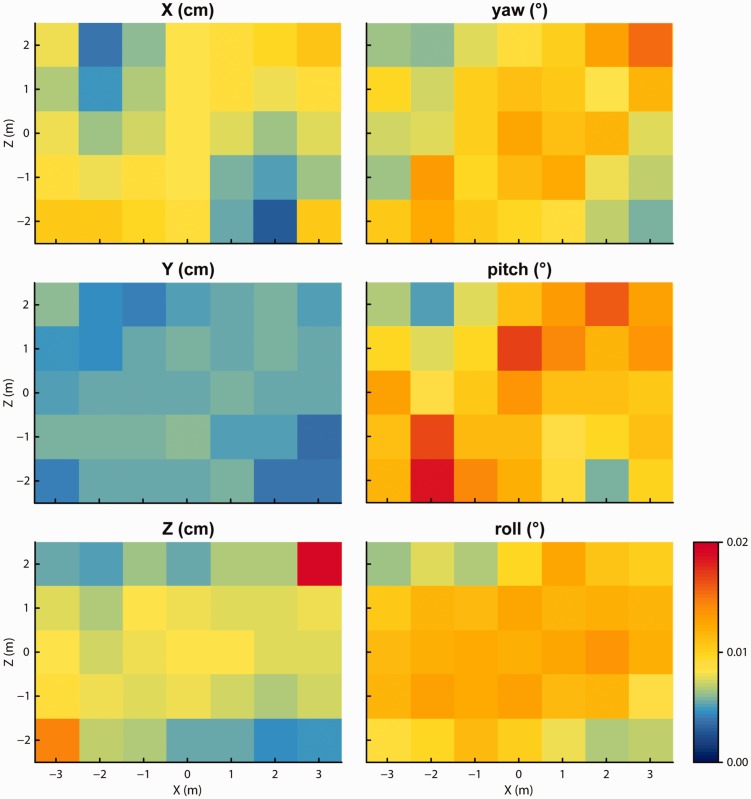
Amount of sample-to-sample jitter (RMS) in each of the reported 3D positions and orientations, at each measurement location in the grid. As there were no systematic differences in RMS between the two headset facing directions, these plots show the data averaged over the two facing directions.

We examined the tracking precision by measuring the size of sample-to-sample jitter in the reported 3D positions and orientations reported by the Vive. The larger the jitter artifacts, the more visible the they become. RMS jitter is plotted for each of the six measurement components in Figure 6, and median values across tracking space are presented in Table 1. The measured RMS levels for each grid point were averaged across the two facing directions because no systematic differences between facing directions were apparent for any of the measurement components. RMS levels were very low, remaining below 0.02 cm and 0.02° in all cases. While this may indicate a low-noise tracking system, the low RMS levels may also be due to filtering in the tracker’s software driver. While for some of the position and orientation measures the RMS levels were lower for the locations closer to the lighthouse units, for other measures the RMS level was mostly constant across the tracking space.

**Table 1.** Median Sample-to-Sample RMS Across Tracking Space for the Three Position and Three Orientation Measures for the Recordings of Tests 2, 4, 5, and 6.

Test	2	4	5	6
System	Vive system 1	Vive system 1	Vive system 2	PPT + InertiaCube
Lighthouse distance (m)	7.45	5	5.66	—
*X* (cm)	0.0080	0.0064	0.0066	0.0173
*Y* (cm)	0.0054	0.0049	0.0052	0.0069
*Z* (cm)	0.0075	0.0055	0.0059	0.0101
Yaw (°)	0.0097	0.0104	0.0045	—
Pitch (°)	0.0111	0.0113	0.0053	—
Roll (°)	0.0114	0.0110	0.0047	—

Values for the orientation measures of the InertiaCube (Test 6) were not available as the InertiaCube apparently provides unchanging values when it is physically static.

**Figure 7.**
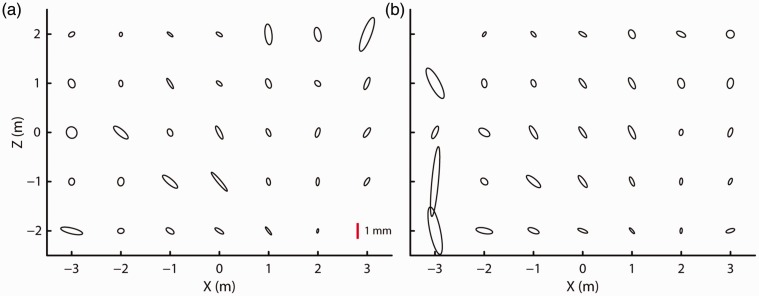
The orientation, aspect ratio, and relative size of the BCEA ellipses at each measurement location for (a) when the headset faced along the positive *X* axis and for (b) when it faced along the negative *X* axis. The red line indicates 1 mm.

Last, to examine the spatial extent of the tracker noise in the reported positions in the *X-Z* plane, we calculated BCEA ellipses for the data recorded at each grid point for both facing directions. These BCEA ellipses, plotted in Figure 7, indicate the size of the area in which 68% of the data points fall and also indicate whether the noise is isotropic or has a larger magnitude along a certain axis. The median area of the BCEA ellipses was 0.025 mm^2^. As shown in the graph, the spatial spread of tracker noise was similar for both facing directions and of similar size for all but the most extreme measurement positions. Furthermore, deviations from isotropy were mostly small except at some positions along the edge of the grid.

### 3. Changing Reference Plane When Regaining Tracking

In Test 1, we observed that systematic offsets occurred in the reported position data after loss of tracking occurred (see Figure 2). We further found that the Vive appeared to perform its measurements with respect to a reference plane that is tilted away from the physical ground plane (see Figure 5). The systematic offsets in reported 3D positions after loss of tracking suggest that the orientation and possibly the position of the Vive’s reference plane changes after the loss and subsequent regaining of tracking. Here we further investigated this issue in a series of three recordings, which simulated the loss and regaining of tracking during situations that increasingly closely resembled what would happen when a participant walks out of the tracking area during an experiment.

#### 3a. Recording 1

In this recording, we placed the Vive at grid location (3, 1) near one of the corners of the tracked area and faced it along the *X* axis of the room. To produce loss of tracking, the Vive was covered for 5 s with a cloth that blocked the infrared light emitted by the lighthouses. The cloth was then removed, and the measurement program watched for tracking being regained. To give the Vive time to stabilize, the measurement program waited for 5 s after tracking was regained before acquiring 2 s of position and orientation data. This procedure was repeated 20 times. We further recorded 1 min of position and orientation data at this location to provide a baseline measure of the variation that occurs due to noise alone.

**Figure 8.**
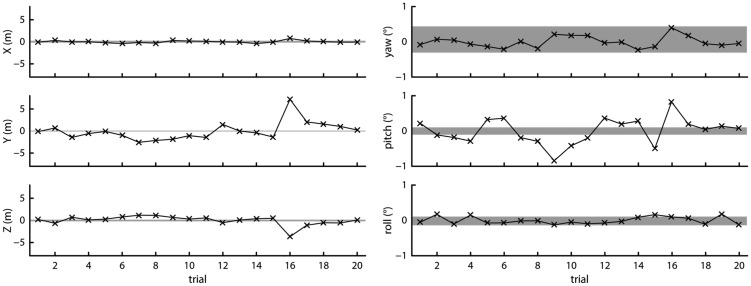
Offsets from the mean measured position (left panels) and orientation (right panels) for 20 trials. The gray shaded areas indicate the range of position or orientation values seen during a 1-min recording.

The left panels of Figure 8 plot the difference of the mean *X*, *Y*, and *Z* positions during each of the 20 measurement trials from the mean observed *X*, *Y*, and *Z* position over all trials. Similarly, the right panels of Figure 8 plot the difference of the mean observed yaw, pitch, and roll orientation during the 20 trials from the mean observed orientations over all trials. The shaded area in all panels indicates the maximum difference from the mean position observed during the 1-min baseline recording. This shaded area indicates the variation in recorded position and orientation that can be expected without intermittent loss of tracking, which varies in extent for the different measures. The plots show that the variation in recorded mean position and orientation along all axes except yaw was larger than what would be expected if the variation was caused by the normally occurring noise in the measurement alone. As the headset’s physical position did not change during the 20 trials, this indicates that after intermittent loss of tracking, the Vive indeed changed its reference plane with respect to which it reports position and orientation measurements. Nevertheless, the observed offsets were small, not exceeding more than a few centimeters or about a degree for the orientation measures.

#### 3b. Recording 2

**Figure 9.**
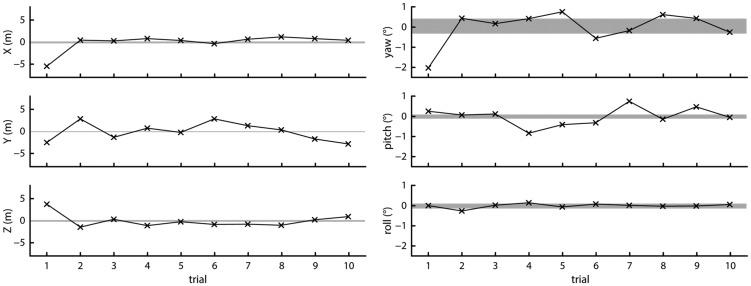
Offsets from the mean measured position (left panels) and orientation (right panels) for 10 trials. The shaded areas indicate the range of position or orientation values seen during a 1-min recording.

The maximum offsets observed in Recording 1 are smaller than those observed in Figure 2. One difference between these two recordings is that when regaining tracking during Test 1, the headset was in another physical location than where it lost tracking. In Recording 1, the headset was in the exact same physical location during all trials. To closer mimic the situation of Figure 2, after covering the headset with a cloth, we moved it to another location before uncovering it and letting it regain tracking. The next trial, it was covered again and moved back to the first location. The two locations used were (3, 1) and (1, 0). In Figure 9, the data for the 10 trials where the headset recovered while at (3, 1) are shown to allow direct comparisons with Recording 1 where the headset was placed at that location for all trials. The plots were made following the same method as for Figure 8, although the scale of the ordinate was increased for the right panels depicting orientation data. Although larger changes in position and orientation are expected from trial to trial as the headset was moved and it was impossible to put it back in exactly the same location, the variation in position and orientation from trial to trial is similar to that observed in Figure 8 for most of the trials. This indicates that regaining tracking in another location than where the Vive lost tracking does not lead to much larger changes of the Vive’s reference plane than when loss and regaining of tracking happen in the same location.

#### 3c. Recording 3

A further difference between Recordings 1 and 2 and Figure 2, as well as a difference between these recordings and a real study situation, is that the headset was not in motion when it regained tracking. In a real study, where a participant wears the Vive, the headset would be moving both when it loses tracking and when it regains tracking. Position and orientation tracking of the Vive depends on a combination of inertial measurement units and the lighthouse optical system, and regaining tracking while moving when both the inertial and the optical systems output time-varying signals may pose a bigger challenge. To mimic this, in this recording we lifted up the Vive after each trial and walked out of the tracking area until it lost tracking. We then walked back in toward the origin of the tracking area and slowly moved the headset from side to side along the *Z* axis until it regained tracking. It was then placed back at position (3, 1), and 2 s of data were recorded after waiting 5 s to ensure the headset was stabilized in its returned position.

**Figure 10.**
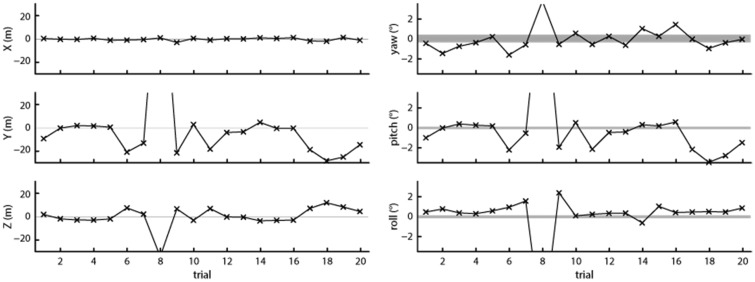
Offsets from the mean measured position (left panels) and orientation (right panels) for 20 trials. The shaded areas indicate the range of position or orientation values seen during a 1-min recording. For Trial 8, the offset was 163 cm along the *Y* axis, −34 cm along the *Z* axis, 15.8° pitch and −11.5° roll.

The results of this recording are plotted in Figure 10, following the same methods and format as in Figures 8 and 9, although the ordinate axis scale was increased. As can be seen from the plots, position and orientation offsets along most axes have become much larger than what was observed in Recordings 1 and 2, despite using the same physical repositioning that led to small offsets in Recording 2. This indicates that when the headset moved while tracking was lost and regained, as would occur in real research situations, the Vive produced large offsets in position and orientation measures, most likely due to large changes in the tilt of the reference plane used for these measurements. As such, a calibration strategy as described in the previous section is not a viable solution to correct for the Vive’s titled reference plane because the calibration procedure would have to be rerun each time after tracking is lost.

### 4. Testing in a Smaller Tracking Area

The findings of unstable tracking reported earlier could be due to the fact that our lighthouse units were placed further apart than the recommended distance of 5.5 m to create a big enough walking space. In Test 4, we therefore placed the lighthouse units closer together and repeated the data measurements made along the grid (Test 2) and after the recovery of tracking while moving (Test 3 c) to test whether the Vive’s tracking became more stable. Specifically, the two lighthouse units were placed at grid locations (−2, 2) and (1, −2) such that they were 5 m apart, and faced directly toward each other. Because the two units now were closer together, they were pointed downward at an angle of 37.5°, which is in the middle of the recommended range. We then reran the Vive’s calibration and tracking space setup procedure, as described earlier.

**Figure 11.**
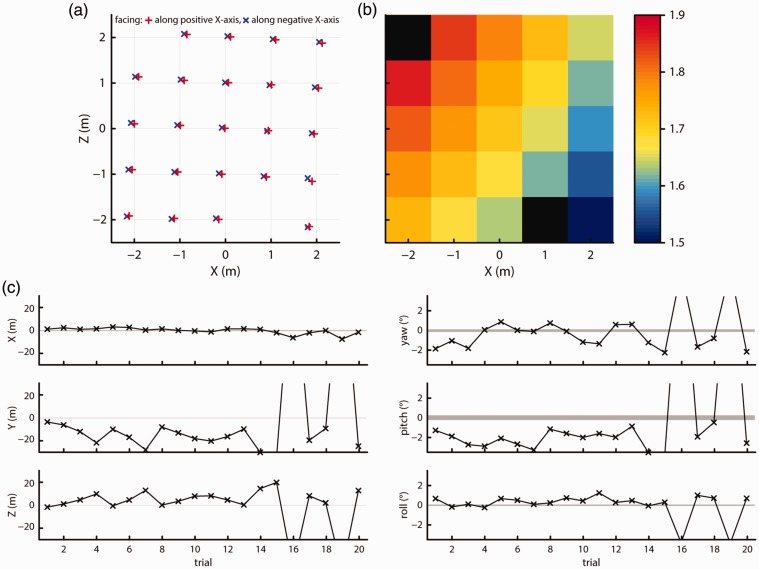
Recorded (a) *X-Z* positions and (b) height at each of the grid locations. No data could be recorded at the black locations due to the loss of tracking. (c) Offsets from the mean measured position (left panels) or orientation (right panels) for 20 trials. The shaded areas indicate the range of position or orientation values seen during a 1-min recording. For Trial 16, the offset was 146 cm along the *Y* axis, −55 cm along the *Z* axis, 6.1° yaw, 19.3° pitch, and −3.8° roll. For Trial 18, the offset was 153 cm along the *Y* axis, −57 cm along the *Z* axis, 6.2° yaw, 18.6° pitch, and −3.9° roll.

Figures 11(a) and 11(b) plot the recorded *X-Z* positions and height above the ground for each grid point for both facing directions. As can be seen in the figure, the *X-Z* measurement positions again fall along a regular and slightly rotated grid, while recorded height varies smoothly and strongly across the space, suggesting a tilted reference plane. RMS noise levels for the position measures decreased by about 10% to 35% from the levels observed in Test 2. RMS noise in the orientation measures however remained unchanged. At 0.016 mm^2^, the median area of the BCEA ellipses has decreased to 65% of the level observed in Test 2. Figure 11(c) shows the mean positions and orientations for 20 trials of loss of tracking and recovery while moving, using the same axes as Figure 10. While the reference noise levels derived from a 1-min recording decreased as indicated by the narrower shaded areas, the variation and magnitude of the recorded position and orientation offsets for each trial are similar to that in Figure 10. In summary, our findings do not change when setting up the Vive tracking system as recommended by the manufacturer.

### 5. Testing a Second Vive System

The findings of unstable tracking reported earlier could be due to a fault in the first Vive system we had acquired. To rule out this possibility, we acquired a second Vive system and used this new headset, controllers, lighthouses, and cables for a new set of measurements. As in Test 4, we repeated the data measurements made along the grid (Test 2) and after the recovery of tracking while moving (Test 3 c). For this measurement, the two lighthouse units directly faced each other, pointed downward at an angle of 37.5°, and were placed at grid locations (−2, 2) and (2, −2) such that they were 5.66 m apart.

**Figure 12.**
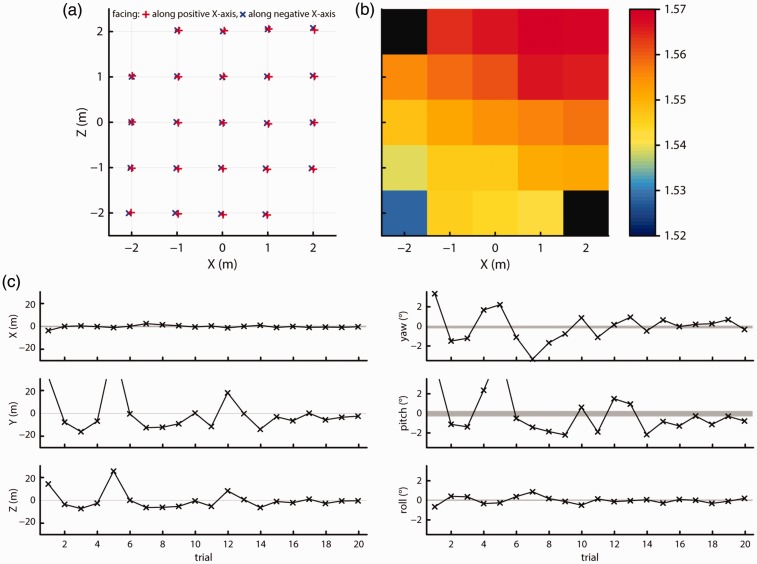
Recorded (a) *X-Z* positions and (b) height at each of the grid locations. No data could be recorded at the black locations due to the loss of tracking. (c) Offsets from the mean measured position (left panels) or orientation (right panels) for 20 trials. The shaded areas indicate the range of position or orientation values seen during a 1-min recording. For Trial 1, the offset was 32 cm along the *Y* axis and 5.2° pitch. For Trial 5, the offset was 59 cm along the *Y* axis and 6.3° pitch.

Figures 12(a) and 12(b) plot the recorded *X-Z* positions and height above the ground for each grid point for both facing directions. As can be seen in the figures, the *X-Z* measurement positions again fall along a regular grid. Recorded height again varied smoothly across the space, although the change in reported height across the tracking space was much smaller at about 4 cm. Nonetheless, the smooth variation in height indicates that this second Vive unit also reports measurements with respect to a tilted reference plane, although the tilt in this system was less than the one we observed in the previous system. Sample-to-sample jitter in the position measurements, as indicated by RMS (Table 1), were the same as the results of the first Vive system reported in Test 4. RMS magnitude of the orientation measures was however half that of the first Vive system. The spatial extent of noise as indicated by BCEA was very similar to that for the first Vive system in Test 4 (median BCEA ellipses area: 0.015 mm^2^).

Figure 12(c) shows the mean positions and orientations for 20 trials of loss of tracking and recovery while moving, using the same axes as Figure 11(c). The reference noise levels derived from a 1-min recording indicated by the shaded areas are the same as for the first Vive unit (cf. Figure 11(c)). Furthermore, the variation and magnitude of the recorded position and orientation offsets for each trial are similar to that of the first Vive system (cf. Figures 10 and 11(c)).

In summary, the reference plane against which this Vive system reports its position and orientation measurements was also tilted away from the physical true ground plane, albeit to a smaller degree. Importantly, with the second Vive system we replicated our finding that the Vive produced large offsets in position and orientation measures after loss of tracking, most likely due to large changes in the tilt of the reference plane used for these measurements.

### 6. Comparison to the WorldViz PPT Tracking System

**Figure 13.**
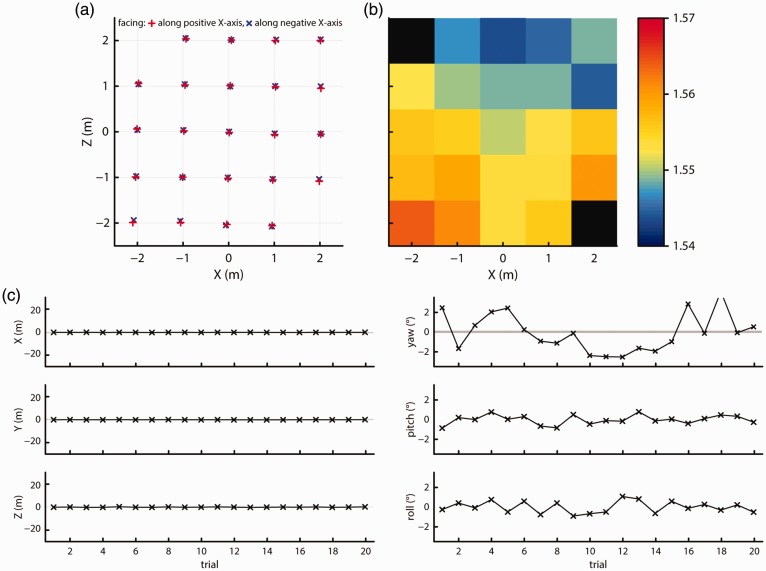
Recorded (a) *X-Z* positions and (b) height at each of the grid locations. No data could be recorded at the black locations due to the loss of tracking. (c) Offsets from the mean measured position (left panels) or orientation (right panels) for 20 trials. The shaded areas indicate the range of position or orientation values seen during a 1-min recording. For Trial 18, the offset was 4.2° yaw.

To compare the Vive’s tracking quality with that of a research-grade position and orientation tracking system, we also acquired data from a research-grade WorldViz PPT-X and InertiaCube system. Its markers were mounted to the same stand as the Vive, which enabled us to acquire this data concurrently with the measurements of the second Vive system reported in Test 5. Figures 13(a) and 13(b) plot the recorded *X-Z* positions and height above the ground for each grid point for both facing directions. As can be seen in the figure, the *X-Z* measurement positions fall along a regular grid, and variations in reported height were random across the tracking space. The maximum difference in reported height between measurement points was about 2 cm. Sample-to-sample RMS jitter (Table 1) in positional measures had a very similar magnitude to that of the Vive for jitter along the *Z* axis and in measured height. RMS along the *X* axis was however double that of the Vive. RMS did not vary systematically over the tracking space for all positional measures. RMS jitter in orientation measures was not available as the InertiaCube apparently provides unchanging values when it is physically static. The spatial extent of noise in the *X-Z* plane, as indicated by a median area of the BCEA ellipses of 0.019 mm^2^, was the same as for the Vive’s data. Furthermore, deviations from isotropy were mostly small.

Figure 13(c) shows the mean position and orientations for 20 trials of loss of tracking and recovery while moving, using the same method as Figure 12(c). In stark contrast to all measurement results from the two Vive systems, offsets in position after loss of tracking never exceeded 0.35 cm for the PPT system, and a large part of these offsets was likely due to variations in placement of the stand. The orientation measures from this system did exhibit variation across trials. The variation in yaw was larger than for pitch and roll, probably because only the variability in yaw was compounded with small placement errors on each trial, whereas pitch and roll would not be affected by such errors. As such, based on the pitch and roll measurements, we estimated intrinsic orientation variation in this system to be about 1.5°. This variation could not have been caused by loss of tracking, as the InertiaCube does not rely on a line-of-sight mechanism that could lead to loss of tracking.

### 7. Examining Vive System Latency

An important characteristic of a VR setup is system end-to-end latency, indicating the time between physical movement of the motion sensor and this movement being reflected on the headset’s screen. If latencies are high, presence is reduced, the virtual environment may appear unstable in 3D space (Jerald & Whitton, 2009) and motion sickness may arise (e.g., Kinsella et al., 2016), for instance, because virtual objects appear to not be static in the world, but slightly move with the observer as s/he moves through the virtual world. To estimate the system latency, we put the Vive on a table in the middle of the tracking area such that the center of its front was 1.55 m above the ground and filmed it from behind with a Casio EX-ZR800 high-speed camera at 480 Hz. In the HMD, we displayed a colorful scene, which was clearly seen from the camera’s view. For each trial, we first collected 4-s data and calculated the mean and standard deviation of the position and orientation values. Then, we sharply pushed the Vive sideways. As soon as any reported position or orientation was outside a threshold defined as 100 × standard deviation from the mean value calculated earlier, the screen was switched to white. From the video recorded at 480 Hz, we estimated the number of screen refreshes the Vive went through at 90 Hz between the headset physically starting to move and the screen turning white. While a threshold of 100 times the standard deviation of naturally occurring noise may seem high, it corresponded to a movement of only 0.12 cm or 0.056° on average, and putting it any lower led to many instances where the system spontaneously crossed the threshold due to noise without it being physically moved. Analysis of six videos in this fashion revealed that it took two refreshes of the Vive’s screen at 90 Hz between the start of the physical movement of the headset and the screen turning white. This corresponds to a system end-to-end latency of about 22 ms. Given our method, this estimate is an upper bound on the true system latency.

## Discussion

An inexpensive HMD and position and orientation tracking system could enable a far larger number of researchers to conduct experiments with moving observers in VR environments. The first commercially available system that has the promise to fulfil this ambition is the HTC Vive. Here, we report tests of its position and orientation tracking capabilities. While tracking is subjectively fast and supports good presence, the system end-to-end latency is low at 22 ms, and the noise level in the tracker output is low, we found that the Vive exhibited two major problems. First, the Vive was shown to use a reference plane that is tilted away from the true ground plane, causing incorrect roll and pitch measurements, as well as changing height measurements across the tracking space. Second, our results suggest that the tilt of this reference plane changed each time the headset lost and regained tracking. These problems were not due to a fault in the first Vive system we acquired, as our results were replicated in the test with a second Vive system. Our observations are reinforced by the feedback discussing the Vive posted on the Internet (e.g., at the online community at www.reddit.com/r/Vive/), where some owners of a Vive system reported that the ground plane in their virtual environments appears to be slanted. The impact of these problems on scientific experiments is discussed below.

In principle, given that we show in this article that the pitch and roll angles reported by the Vive are consistent with its tilted reference plane, one could measure the tilted reference plane used by the Vive once before each experimental run, and correct for it with a calibration procedure. However, our results and personal experience with the system indicate that the plane orientation may change when the Vive briefly loses tracking (e.g., when a participant walks out of the tracking area or puts one’s hands in front of the Vive’s sensors). These offsets after tracking is lost and regained were found to be random from one trial to the next and did not increase with the number of times tracking was lost. This makes a calibration approach to correcting for the Vive’s tilted reference plane difficult in practice, as it would need to be repeated each time after the Vive loses tracking.

A slanted ground plane and changing eye-height across the tracking space pose a problem for many studies of the freely moving observer. A slanted ground plane and incorrect eye-height measurements would be fatal for, for example, studies of human locomotion, where perception and action are understood to occur in units of eye-height (see e.g., Warren & Whang, 1987). Other examples of studies for which these problems are fatal are the study of the perception of travel distance (Frenz & Lappe, 2005) or the investigation of cue use during walking through systematic manipulations of optic flow (e.g., Li et al., 2014; Warren et al., 2001). The changes in optic flow rates that occur across a tilted ground plane and as eye-height changes are likely to affect the use of optic flow information and would thus invalidate the results of these experiments.

However, for other classes of experiments, these imperfections in the Vive’s tracking output may not pose a problem. For instance, one can likely use the Vive for experiments in which the risk of losing tracking is small because the participant only moves in a small area. When loss of tracking is unlikely, a single calibration before an experiment run may enable collecting data of sufficient quality. Furthermore, experiments where a few degrees of offset in pitch and yaw measurements do not matter can be run, as well as experiments where the researcher does not use all of the tracker’s measurements, for example, displays simulating self-motion through a virtual environment that use only the yaw measure of the Vive to allow the participant to look around during the simulated self-motion.

In summary, we are excited to see the developments in the VR market that may make VR equipment available to a larger number of researchers and look forward to what the future may bring. The Vive has already brought needed innovations in terms of the controller, the front camera, and the display with its high resolution, large field of view, and high refresh rate. Furthermore, multiple new systems have been announced in recent months, and the manufacturers of these and already released systems are working relentlessly on new and improved versions. We hope however that our results show the potential caveats to researchers considering using these consumer-level VR systems. While commercial systems may serve a game playing enthusiast well, they will not necessarily meet the requirements of scientific research. It is therefore important to carefully examine a VR system and judge whether it is suitable for the purposes of one’s experiment. To this end, we have made our data acquisition and analysis code freely available at http://dx.doi.org/10.5281/zenodo.569884. We encourage researchers using consumer-level VR systems to report the results of such tests in their articles, to substantiate that the quality of their setup was sufficient for the purposes of their study.
